# Dual Phosphoinositide 3-Kinase/Mammalian Target of Rapamycin Inhibitor NVP-BEZ235 Has a Therapeutic Potential and Sensitizes Cisplatin in Nasopharyngeal Carcinoma

**DOI:** 10.1371/journal.pone.0059879

**Published:** 2013-03-22

**Authors:** Fen Yang, Xiao-Jun Qian, Wei Qin, Rong Deng, Xiao-Qi Wu, Juan Qin, Gong-Kan Feng, Xiao-Feng Zhu

**Affiliations:** 1 State Key Laboratory of Oncology in South China, Sun Yat-Sen University Cancer Center, Guangzhou, Guangdong Province, China; 2 Key Laboratory of Human Functional Genomics of Jiangsu Province, Department of Biochemistry and Molecular Biology, Nanjing Medical University, Nanjing, Jiangsu Province, China; University of South Alabama, United States of America

## Abstract

Phosphoinositide 3-kinase (PI3K)/AKT/mammalian target of rapamycin inhibitor (mTOR) pathway is often constitutively activated in human tumor cells and thus has been considered as a promising drug target. To ascertain a therapeutical approach of nasopharyngeal carcinoma (NPC), we hypothesized NVP-BEZ235, a novel and potent imidazo[4,5-c] quinolone derivative, that dually inhibits both PI3K and mTOR kinases activities, had antitumor activity in NPC. Expectedly, we found that NVP-BEZ235 selectively inhibited proliferation of NPC cells rather than normal nasopharyngeal cells using MTT assay. In NPC cell lines, with the extended exposure, NVP-BEZ235 selectively inhibited proliferation of NPC cells harboring PIK3CA mutation, compared to cells with wild-type PIK3CA. Furthermore, exposure of NPC cells to NVP-BEZ235 resulted in G1 growth arrest by Propidium iodide uptake assay, reduction of cyclin D1and CDK4, and increased levels of P27 and P21 by Western blotting, but negligible apoptosis. Moreover, we found that cisplatin (CDDP) activated PI3K/AKT and mTORC1 pathways and NVP-BEZ235 alleviated the activation by CDDP through dually targeting PI3K and mTOR kinases. Also, NVP-BEZ235 combining with CDDP synergistically inhibited proliferation and induced apoptosis in NPC cells. In CNE2 and HONE1 nude mice xenograft models, orally NVP-BEZ235 efficiently attenuated tumor growth with no obvious toxicity. In combination with NVP-BEZ235 and CDDP, there was dramatic synergy in shrinking tumor volumes and inducing apoptosis through increasing Noxa, Bax and decreasing Mcl-1, Bcl-2. Based on the above results, NVP-BEZ235, which has entered phase I/II clinical trials in patients with advanced solid tumors, has a potential as a monotherapy or in combination with CDDP for NPC treatment.

## Introduction

Nasopharyngeal carcinoma (NPC) is the most common cancer in certain regions of East Asia and Africa, caused by the synergetic effect of Epstein-Barr virus (EBV) infection, genetic aberrations, environmental and dietary factors, especially in males [1], [2]. Although early-stage tumors are sensitive to radiotherapy, patients with advanced NPC tend to experience therapy failure due to the highly invasive and metastatic nature of the disease. Cisplatin (CDDP)-based combination chemotherapy is regarded as the most effective regimen for metastatic NPC, but the efficacy of CDDP for treating NPC is limited due to dose-related toxicity and resistance.

Most of NPC are driven by the accumulation of genetic and epigenetic alterations [3], which leads to synergistic interaction from a complex of signal transduction processes, including multiple onco-proteins and tumor suppressors such as Ras, Myc, phosphatidylinositol 3-kinase (PI3K)/AKT/mammalian target of rapamycin inhibitor (mTOR), HER2/Neu, P53 and phosphatase and tensin homolog deleted on chromosome Ten (PTEN). Specifically, PI3K/AKT and mTOR pathways have been shown to play pivotal roles in tumor growth as they promote cell mass increase and cell cycle entry, counteract apoptosis, modulate cytoskeletal rearrangements, and enhance cell migration [4], [5]. Therefore, it is critical to examine therapeutic agents that explicitly target both the PI3K/AKT and mTOR signalling cascades in diseases, such as NPC, that harbor the activation of the PI3K/AKT pathway. The PIK3CA gene at 3q26.32 was found to be one of the candidate oncogenes, and amplification and overexpression of PIK3CA were frequently detected in NPC. PIK3CA encodes the p110 catalytic subunit of PI3K which is involved in the cell signaling through catalysing the production of the phosphatidylinositol 3,4,5-triphosphate (PIP3) from phosphatidylinositol 4,5 bisphosphate (PIP2) [6]. PIK3CA mutations were discovered in a large-scale mutational analysis in various cancers, including 25–30% of colorectal cancers, gastric cancers and brain tumors [7], [8]. Furthermore, *in vitro* studies demonstrated that the PIK3CA mutant (H1047R) had increased kinase activity, which is a gain-of-function mutation [9].

NVP-BEZ235 is an imidazo[4,5-c]quinoline derivative that inhibits PI3K and mTOR kinases activities by binding to the ATP-binding cleft of these enzymes [10]. It is an ATP-competitive pan-class I PI3K inhibitor that is effective against p110a with hotspot mutations, and likewise inhibits both mammalian target of rapamycin complex 1 (mTORC1) and mTORC2 [10]–[12]. NVP-BEZ235 has entered phase I/II clinical trials in patients with advanced solid tumors and showed higher efficacy in cancers with PIK3CA mutant. NVP-BEZ235 was reported to strongly reverse the effect of hyperactivation of the PI3K pathway by either loss of PTEN function or by activation of PI3K mutations, which is resistant to lapatinib [11].

Combined modality treatment using concurrent CDDP-based chemotherapy is so far the only strategy supported by several large randomized studies to improve survival for NPC [13], [14]. However, treatment of cancers by CDDP often results in the development of resistance, including NPC. This study aimed to investigate the antitumor effect and chemosensitization of PI3K/mTOR inhibitor NVP-BEZ235 in NPC both *in vivo* and *in vitro*, which would gain more insight on the potential of using NVP-BEZ235 to treat NPC and overcome the resistance of CDDP therapy, especially in NPC harboring PIK3CA mutations.

## Materials and Methods

### Ethics Statement

This study and experimental protocols were approved by the Institutional Animal Care and Use Committee of Sun Yat-Sen University Cancer Center with permit number 10211002C.

### Cell Lines, Antibodies and Reagents

The human NPC cell lines CNE1, CNE2, HONE1, SUNE1 and normal nasopharyngeal cells NP69 are cultured and conserved by Sun Yat-sen University and have been published before [15]–[18]. Cancer Center and cultivated in Dulbecco’s modified Eagle’s medium supplemented with 10% fetal bovine serum in a humidified atmosphere containing 5% CO_2_ at 37°C. AKT, phospho-AKT (Ser473), phospho-AKT (Thr308), glycogen synthetase kinase-3 α/β (GSK3α/β) and Caspase-3, poly (ADP-ribose) polymerase (PARP), ERK1/2, phospho-ERK1/2 (Thr202/Tyr204) and horseradish peroxidase–conjugated secondary antibodies were purchased from Santa Cruz Biotechnology (California, U.S.A.). Antibodies against 4E-binding protein l (4E-BPl), mTOR, ribosome S6 kinase (S6K), proline-rich AKT substrate of 40 KD (PRAS40), phospho-4E-BP1 (Thr37/46), phospho-mTOR (Ser2448), phospho-S6K (Thr389), phospho-PRAS40 (Thr246) and phospho-GSK3β (Ser9) were obtained from Cell Signaling Technology (Boston, U.S.A.). Propidium iodide (PI), DMSO and MTT were purchased from Sigma-Aldrich (St. Louis, U.S.A.).

### Chemical Compounds and Drugs

BEZ235 and U0126 were obtained from Selleck Chemical in U.S.A., and provided as a 10 mM stock solution in 100% DMSO and stored at −20°C. CDDP solution was purchased from Mayne Pharma Pty. Ltd and stored at 4°C. Working solutions were prepared freshly before addition to the cell media. Final DMSO concentration was kept constant at 0.1% in control and compound-treated cells.

### Oncogenic Mutation Profiling Detected with the Sequenom Platform

The OncoCarta Panel v1.0 and MassARRAY System (Sequenom, U.S.A.) were used to mutation screens according to the protocol provided by Sequenom. This system involves PCR amplification of genomic DNA, followed by primer extension, and mass spectrometry (MALDI-TOF MS). Briefly, genomic DNA was amplified using the supplied OncoCarta PCR primers, unincorporated nucleotides were inactivated by shrimp alkaline phosphatase (SAP), and a single base extension reaction was performed using extension primers. Salts were removed by Clean Resin (Sequenom, U.S.A.), and multiplexed reaction solution was dispensed onto a 384 format SpectroChipII matrix chips using the MassARRAY Nanodispenser RS1000 (Sequenom, U.S.A.). Mass spectrometry analysis was performed on a matrix-assisted laser desorption/ionization time of flight spectrometer (Sequenom, U.S.A.), with data analysis performed MassArray Typer Analyzer software 4.0.4.20 (Sequenom, U.S.A.).

### Cell Proliferation by MTT Assay

Cells were treated with different concentrations of NVP-BEZ235 for indicated time. The method has been described elsewhere [19]. Absorbance values were measured at the wavelength of 570 nm. Inhibitory rates were calculated by Microsoft Excel and IC50 values were calculated using the Calcusyn software.

### Cell Cycle and Apoptosis by PI Uptake Assay

Cells were seeded at 0.5∼1×10^5^ ****per well in 24-well plates and allowed to reach exponential growth for 16**∼**24 hours before treatment. PI staining procedure followed the instruction’s protocol which was performed as described elsewhere [20]. PI buffer was mixed with 100 ml dH_2_O, 0.005 g PI, 0.1 g Trisodium Citrate, 0.1 ml Triton X-100, covering with foil and keeping at 4°C. Cell cycle and apoptosis were quantified by measuring the DNA content of cells by flow cytometry, which the proportion of sub-G1 phase cells (%) represented the apoptotic rate and proportions of other phases cells (%) reflected cell cycle.

### Proteins Extraction and Western Blotting

The method has also been described elsewhere [19]. Briefly, cells were solubilized in 1×lysis buffer (20×lysis buffer, Cell Signaling Technology, U.S.A.) 30 mins and centrifuged at 4°C, 12000 rpm. The protein concentrations were determined using the Pierce BCA protein assay kit. Proteins were separated electrophoretically in SDS-polyacrylamide gels and transferred to PVDF membranes. The membranes were then blocked in the 5% skim milk solution in Tris-buffered saline containing 0.1% Tween (TBST) for 2 h and then incubated at 4°C overnight with the primary antibody, and subsequently incubated with the HRP-conjugated secondary antibody at room temperature for 2 h. Immunoreactivity was detected using the Amersham ECL Prime Western blotting detection reagent (GE Healthcare, U.S.A.) according to the manufacturer’s instructions.

### Establishment of Xenograft Tumors and *in vivo* Study

Animal studies were performed in accordance with the criteria outlined in the "Guide for the Care and Use of Laboratory Animals" prepared by the National Academy of Sciences and published by the National Institutes of Health (U.S.A.). Male and female BALB/C nude mice were purchased from Hunan Silaikejingda Laboratory Animal Technology Co. Ltd (P.R.C.). The 5-week-old BALB/C nude mice used were housed in laminar flow cabinets under specific pathogen-free conditions. A total of 4×10^6^ (0.2 ml) human NPC cells per mouse were inoculated subcutaneously into the right dorsal flanks of nude mice. When tumors reached an average volume of approximately 0.1 cm^3^, the mice were randomized into control and treated groups (n  = 6 per group). The treatment groups received doses of 25 mg/kg NVP-BEZ235, 50 mg/kg NVP-BEZ235, 2.5 mg/kg CDDP, 25 mg/kg NVP-BEZ235 combined with 2.5 mg/kg CDDP and 50 mg/kg NVP-BEZ235 combined with 2.5 mg/kg CDDP. The negative control groups received vehicle only, i.e., normal saline (NS) and solvent (N-Methylpyrrolidone: Polyethylene Glycol 300 = 1∶9). NVP-BEZ235 and solvent were administered by intragastric administration once daily for 5 consecutive days each week for up to 19∼21 days. CDDP and NS were administered by intraperitoneal injection once per 4 days for up to 19∼21 days. Tumor sizes were measured by caliper and recorded every 3 days. The tumor volumes were calculated from the length (the longest diameter across the tumor) and width (the corresponding perpendicular diameter) using the following formula: volume = π/6×length×width^2^. The tumor growth inhibitory rates were calculated as 100%×(1−average size of treated tumor/size of control tumor) on each measurement day. Animal body weight was measured and recorded every 2∼3 days during the treatment. On the day following the last dose, animals were euthanized and tumors were resected, preserved at −80°C for Western blotting.

### Statistical Analysis

The data were expressed as the means and standard errors. Statistical analysis was performed with the two-tailed Student’s t test and ANOVA, in which *P*<0.05 was considered significant.

## Results

### NVP-BEZ235 Selectively Inhibited Proliferation of NPC Cells Harboring PIK3CA Mutation

**Figure 1:**
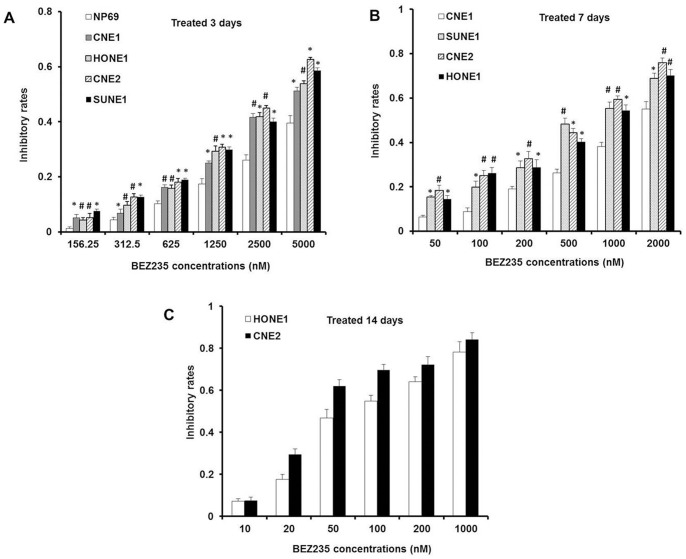
NVP-BEZ235 inhibited cell proliferation in NPC cells. (A) Inhibitory effects of NVP-BEZ235 on NPC cells and normal nasopharyngeal cells. Cells were cultured at 8,000∼10,000 cells/well in 96-well plates and cell growth was assessed by MTT methods after 3 days of treatment with NVP-BEZ235 (0–5000 nM).The results were expressed as inhibitory rates of cells. n = 3,* *P*<0.05, **#** *P*<0.01, inhibitory rates of the corresponding concentration from NPC cells *vs.* NP69 cells. The inhibitory rate was calculated by following formula: A =  (1-X/Y) ×100%. A, inhibitory rate; X, the average absorbance values of experimental groups; Y, the average absorbance values of control groups. (B) Inhibitory effects of NVP-BEZ235 on NPC cells with treatment for 7 days. Cells were cultured at 2,000∼4,000 cells/well in 96-well plates and cell growth was assessed by MTT methods after 7 days of treatment with NVP-BEZ235 (0–2000 nM). * *P*<0.05, **#** *P*<0.01, inhibitory rates of the corresponding concentration from other NPC cells *vs.* CNE1 cells. (C) Inhibitory effects of NVP-BEZ235 on CNE2 and HONE1 cells with treatment for 14 days. Cells were cultured at 500∼1,000 cells/well in 96-well plates and cell growth was assessed by MTT methods after 14 days of treatment with NVP-BEZ235 (0–1000 nM).

Oncogenic mutation profiling detected with the Sequenom platform showed that the missense mutation PIK3CA (H1047R) was identified in 3/4 NPC cell lines CNE2, HONE1 and SUNE1 (Table 1, Figure S1). To determine the effect of NVP-BEZ235 on cell proliferation, we treated CNE1, CNE2, HONE1 and SUNE1 cells and normal nasopharyngeal cells NP69 with different concentrations of NVP-BEZ235 for 3 days and found that it selectively inhibited proliferation of NPC cells, compared to NP69 (Fig. 1A). The inhibitory assay was performed over 3 days, but did not necessarily reflect the efficacy of different treatments over longer periods of time. Thus, we performed the long-term treatment over 7 days to further evaluate the potencies of NVP-BEZ235 on NPC cells, and found that there were different inhibitory effects between PIK3CA wild-type CNE1 cells and PIK3CA mutant cells. As shown in Fig. 1B, CNE1 cells with NVP-BEZ235 treatment also displayed increased inhibitory rates, but substantially less effective than that of the PIK3CA mutant CNE2, HONE1 and SUNE1 cells, which IC50 values were 1913, 493, 699 and 667 nM, respectively. As to PIK3CA mutant cells, with the extended exposure, NVP-BEZ235 treatment induced similar inhibitory effect only at lower concentration. IC50 values for NVP-BEZ235 treatment after 14 days were 86.9 and 39.7 nM in CNE2 and HONE1 cells, respectively (Fig. 1C).

**Table 1:** Oncomutation panel mutation list report.

Sample	Gene	Assay	Mutation	Allele	Chip: Well	WTFrequency	Mutation Frequency	Z-score	Confidence	Notes
CNE1	HRAS	HRAS_3	G13R	G	G0582620_(1) (1) : M17	0.807	0.193	10	1.High	T
CNE1	HRAS	HRAS_3	G13R	G	CUSTOMER	0.81	0.19			T
CNE2	HRAS	HRAS_3	G13R	G	G0582620_(1) (1) : N17	0.753	0.218	10	1.High	T
CNE2	HRAS	HRAS_3	G13R	G	CUSTOMER	0.75	0.22			U
CNE2	PIK3CA	PIK3CA_9	H1047R	G	G0582620_(1) (1) : N02	0.363	0.637	10	1.High	T
CNE2	PIK3CA	PIK3CA_9	H1047R	G	CUSTOMER	0.45	0.55			T
HONE1	HRAS	HRAS_3	G13R	G	G0582620_(1) (1) : O17	0.796	0.204	7.98	1.High	T
HONE1	HRAS	HRAS_3	G13R	G	CUSTOMER	0.8	0.2			T
HONE1	PIK3CA	PIK3CA_9	H1047R	G	G0582620_(1) (1) : O02	0.497	0.503	10	1.High	T
HONE1	PIK3CA	PIK3CA_9	H1047R	G	CUSTOMER	0.5	0.5			T
SUNE1	PIK3CA	PIK3CA_9	H1047R	G	G0582620_(1) (1) : P02	0.484	0.516	10	1.High	T
SUNE1	PIK3CA	PIK3CA_9	H1047R	G	CUSTOMER	0.48	0.52			T

### NVP-BEZ235 Dually Inhibited PI3K/AKT and mTORC1 Pathways in NPC Cells with PI3KCA Mutation

**Figure 2:**
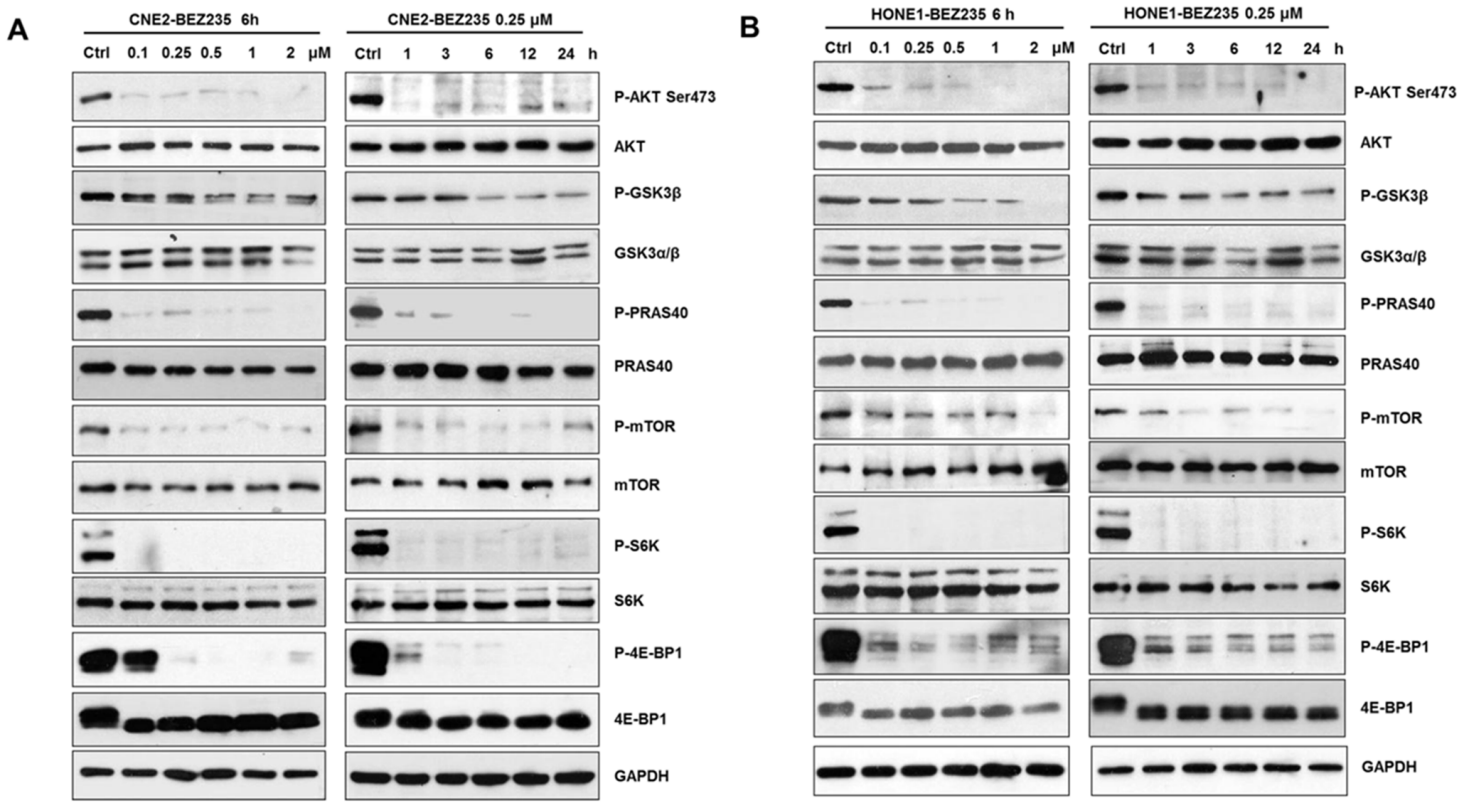
NVP-BEZ235 inhibited activities of PI3K/AKT and mTORC1 pathways. (A) (B) NVP-BEZ235 blocked the phosphorylation of AKT, GSK3β, PRAS40, mTOR, S6K and 4E-BP1 in CNE2 and HONE1 cells. Cells were cultured at 2∼4×10^5^cells/well in 6-well plates and were treated with NVP-BEZ235 0.25 µM in indicated periods and different concentrations for 6 h and analyzed by Western blotting.

We investigated whether NVP-BEZ235 could inhibit the phosphorylation of downstream targets of PI3K/AKT and mTORC1 pathways in CNE2 and HONE1cells harboring PIK3CA mutation. It is well known that the phosphorylation of AKT and its substrates glycogen GSK3β and PRAS40 directly reflects PI3K and AKT kinases activities, the phosphorylation of 4E-BPl and S6K represents mTORC1 kinase activity. As expected, the phosphorylation of AKT, GSK-3β, PRAS40, S6K and 4E-BP1 was markedly reduced by NVP-BEZ235 but the total proteins of them were not affected in CNE2 and HONE1cells (Fig. 2), which was consistent with studies of other tumors, such as breast cancer, gliomas and myeloma [12], [21], [22].

### NVP-BEZ235 Induced Cell Cycle Arrest in G1 Phase through Regulating Cell Cycle Related Proteins

**Figure 3:**
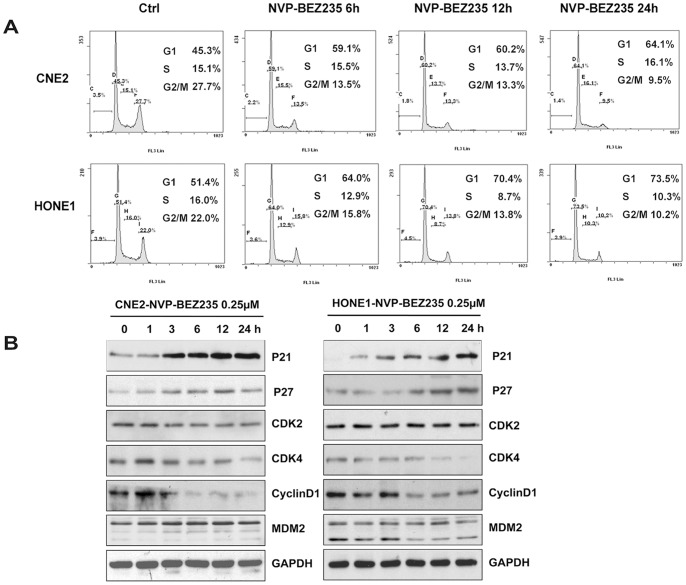
NVP-BEZ235 induced cell cycle arrest in G1 phase by regulating cycle related proteins. (A) NVP-BEZ235 increased G1 phase cells population. Cells were cultured at the density of 0.5∼1×10^5^ cells/well in 24-well plates and were treated with NVP-BEZ235 0.25 µM in indicated periods by PI staining. *P*<0.05, G1 phase cells rates of each period *vs.* control cells, both in CNE2and HONE1 cells. (B) NVP-BEZ235 decreased CDK4, CyclinD1 and increased P21, P27 in protein levels. Cells were cultured at 2∼4×10^5^cells/well in 6-well plates and were treated with NVP-BEZ235 0.25 µM in indicated periods and analyzed by Western blotting.

When treating cells with NVP-BEZ235 0.25 µM for different time periods, we found that G1 phase cells increased in a time-dependent manner (Fig. 3A), which both CNE2 and HONE1 cells played significant differences compared to untreated cells. It is well reported that the PI3K/AKT pathway plays a key role in G1 phase cell cycle, which AKT activation increases the transcription of c-Myc, a strong promoter of cell cycle which causes cells to exit G0 both by inducing D-type cyclins and suppressing negative regulators P27, P21, and P15 [23], [24]. AKT also indirectly controls the stability of c-Myc and cyclin D1 via its downstream substrate GSK-3β [25], [26]. Here NVP-BEZ235 decreased CDK4 and CyclinD1, and increased P21 and P27 in protein levels by inhibiting AKT activity, but not for MDM2 and CDK2 (Fig. 3B).

### NVP-BEZ235 Alleviated the Activation of PI3K/AKT and mTORC1 Pathways by CDDP

**Figure 4:**
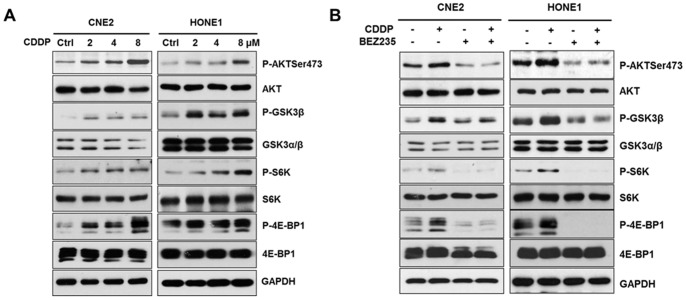
NVP-BEZ235 alleviated the activation of PI3K/AKT and mTORC1 pathways by CDDP. (A) CDDP increased the phosphorylation of downstream substrates of PI3K/AKT and mTORC1 pathways. Cells were cultured at 2∼4×10^5^cells/well in 6-well plates and were treated with CDDP (0∼8 µM) for 6 h and analyzed by Western blotting. (B) NVP-BEZ235 alleviated the phosphorylation downstream substrates of PI3K/AKT and mTORC1 pathways by CDDP. Whole cell lysates from CNE2 and HONE1 cells pretreated with NVP-BEZ235 (0.25 µM) for 1 h with the addition of CDDP for 6 h and analyzed by Western blotting.

CDDP-resistance frequently leads to poor prognosis and the associated mechanisms include JNK pathway activation, P38MAPK activation, EGFR activation and AKT activation [27]–[30]. To investigate CDDP resistant mechanism in NPC, we treated cells with CDDP and found that the phosphorylation of AKT, GSK-3β, S6K and 4E-BP1 increased in dose-dependent manners, which suggested that activation of PI3K/AKT and mTORC1 pathways was induced by CDDP (Fig. 4A). When cells were pretreated with NVP-BEZ235 for 1 h and then treated with CDDP, the phosphorylation of AKT, GSK-3β, S6K and 4E-BP1 was markedly reduced, which indicated that NVP-BEZ235 alleviated the activation of PI3K/AKT and mTORC1 pathways by CDDP (Fig. 4B).

### CDDP and NVP-BEZ235 Synergistically Inhibited Proliferation and Induced Apoptosis

**Figure 5:**
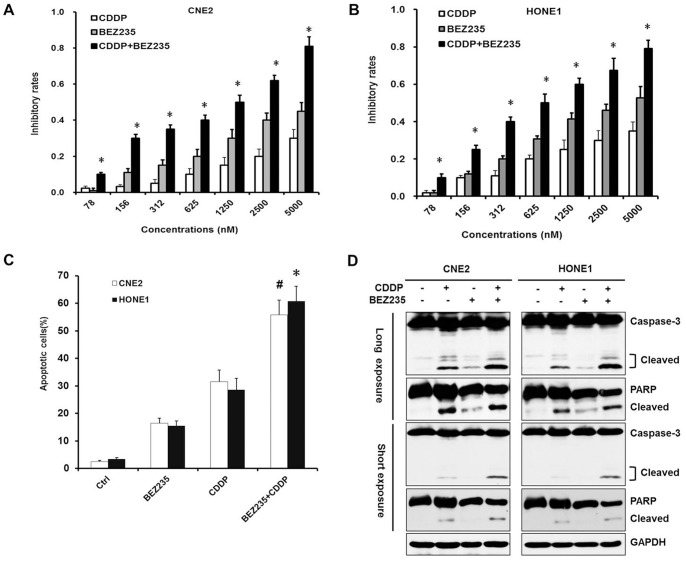
CDDP and NVP-BEZ235 synergistically inhibited NPC cells proliferation and induced apoptosis. (A) (B) Combination of CDDP and NVP-BEZ235 synergistically inhibited cell proliferation. Cells were cultured at 8,000–10,000 cells/well in 96-well plates and cell growth was assessed by MTT methods with treatment of CDDP, NVP-BEZ235 and combination of NVP-BEZ235 and CDDP, respectively. *P<0.05, combination compared to CDDP alone and NVP-BEZ235 alone, respectively. (C) Combination of CDDP and NVP-BEZ235 synergistically induced apoptosis. Cells were cultured at 0.5∼1.0×10^5^cells/well in 24-well plates and apoptosis was assessed by PI staining methods with treatment of CDDP, NVP-BEZ235 and combination of NVP BEZ235 and CDDP, respectively. # *P*<0.05 (CNE2), *P<0.05 (HONE1), combination *vs.* CDDP alone and NVP-BEZ235 alone, respectively. (D) Combination of CDDP and NVP-BEZ235 synergistically induced cleavages of Caspase-3 and PARP. Cells were cultured at 2.0∼4.0×10^5^cells/well in 6-well plates and were treated with CDDP, NVP-BEZ235 and combination of NVP-BEZ235 and CDDP, respectively, and analyzed by Western blotting.

NVP-BEZ235 alleviating the activation of PI3K/AKT and mTORC1 pathways by CDDP partly resolved CDDP resistance. Inhibitory rates from combination of CDDP and NVP-BEZ235 were significantly higher than CDDP alone or NVP-BEZ235 alone and combination indexes (CI) were all less than 1 in a series of concentrations (Fig. 5A and B). As shown in Fig. 5C, apoptotic cells from combination of CDDP and NVP-BEZ235 were also markedly higher than CDDP alone or NVP-BEZ235 alone. Apoptosis related proteins PARP and Caspase-3 from combination of CDDP and NVP-BEZ235 were obviously cleaved, cleavages showed significant increase, compared to CDDP alone and NVP-BEZ235 alone (Fig. 5D). These data suggested that NVP-BEZ235 sensitized CDDP-induced proliferation inhibition and apoptosis.

### Antitumor Activity of NVP-BEZ235 and its Synergy with CDDP in Nude Mice Xenografts

**Figure 6:**
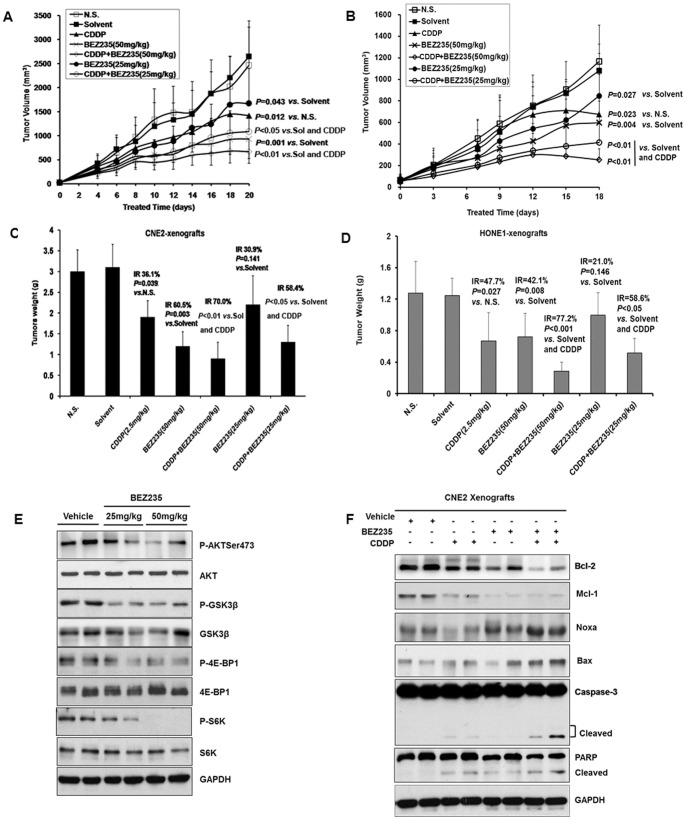
Antitumor effect of NVP-BEZ235 and its synergy with CDDP *in vivo.* (A) (B) The antitumor efficacies of NVP-BEZ235, CDDP and combination of NVP-BEZ235and CDDP in CNE2 and HONE1 tumor xenografts by calculating tumor volumes. (C) (D) Antitumor efficacies of NVP-BEZ235 and CDDP *in vivo* by calculating tumor weights. Nude mice were killed and tumors were intactly isolated to weight every tumor tissue. (E) NVP-BEZ235 inhibited downstream substrates of the PI3K/AKT and mTORC1 pathways *in vivo*. Whole cell lysates from tumor samples were subjected to Western blotting. The proteins were randomly extracted from two xenograft tumors from two mice in each group. (F) NVP-BEZ235and CDDP synergistically induced apoptosis. Apoptosis related proteins were detected by Western blotting.

To investigate the antitumor activity of NVP-BEZ235 and its synergy with CDDP *in vivo*, we established CNE2 and HONE1 xenografts in nude mice and found that they were consistent with results of the *in vitro* experiments. Tumor growth inhibitory rates of treatment groups were significantly higher than the control and inhibitory rates of NVP-BEZ235 in combination with CDDP were significantly higher than NVP-BEZ235 alone or NVP-BEZ235 alone. According to tumor volumes of CNE2 xenografts, tumor growth inhibitory rates were 36.9% and 64.7% in the 25 mg/kg and 50 mg/kg NVP-BEZ235 groups, respectively. Furthermore, NVP-BEZ235 in combination with CDDP synergistically suppressed tumor growth in xenograft models. The inhibitory rates of CDDP 2.5 mg/kg in combination with NVP-BEZ235 25 mg/kg or 50 mg/kg were 59.1% and 74.7% (Fig. 6A). In the HONE1 xenografts, inhibitory rates from tumor volumes were 21.6% and 44.6% in the 25 mg/kg and 50 mg/kg NVP-BEZ235 groups, respectively. The inhibitory rates of CDDP 2.5 mg/kg in combination with NVP-BEZ235 25 mg/kg or 50 mg/kg were 61.6% and 76.5% (Fig. 6B). According to tumor weight of CNE2 xenografts, the tumor growth inhibitory rates were 30.9% and 60.5% in the 25 mg/kg and 50 mg/kg NVP-BEZ235 groups, respectively. The inhibitory rates of CDDP 2.5 mg/kg in combination with NVP-BEZ235 25 mg/kg or 50 mg/kg were 58.4% and 70.0% (Fig. 6C). In the HONE1 xenografts, inhibitory rates from tumor weight were 21.0% and 42.1% in the 25 mg/kg and 50 mg/kg NVP-BEZ235 groups, respectively. The inhibitory rate of CDDP 2.5 mg/kg in combination with NVP-BEZ235 25 mg/kg or 50 mg/kg were 58.6% and 77.2% (Fig. 6D).

Furthermore, in Figure S2, body weights of nude mice from CNE2 xenografts had no significant differences for treatment groups compared to the control, which showed that NVP-BEZ235 25 mg/kg and 50 mg/kg groups had no any toxicity for CNE2 xenografts treatment. However, in the HONE1 xenografts, CDDP group, CDDP in combination with NVP-BEZ235 25 mg/kg and 50 mg/kg, body weights of nude mice were significantly decreased compared to the control, but not in the 25 mg/kg and 50 mg/kg NVP-BEZ235 groups. The data suggested that CDDP treatment caused severe toxicity and combination with NVP-BEZ235 did not reduce the toxicity. Seeing that CDDP in combination with NVP-BEZ235 got synergic effects, NVP-BEZ235 could replace CDDP in treating NPC as a new therapeutical approach, otherwise we should reduce CDDP dose in combined strategy to alleviate the toxicity.

The effect of NVP-BEZ235 on the AKT signaling pathway *in vivo* was consistent with results of the *in vitro* experiments. NVP-BEZ235 dramatically suppressed the phosphorylation of AKT, GSK3β, S6K and 4E-BP1 (Fig. 6E). The combination of CDDP and NVP-BEZ235 synergistically increased levels of pro-apoptotic proteins Bax and Noxa and decreased levels of anti-apoptotic proteins Mcl-1 and Bcl-2. Furthermore, the combination markedly induced cleavages of caspase-3 and PARP. These data suggested that the combination of NVP-BEZ235 with CDDP synergistically induced apoptosis *in vivo* through regulating Bcl-2 family numbers (Fig. 6F).

## Discussion

NPC is different from other head and neck cancers for its unique epidemiology, natural biological behavior and therapeutic considerations. Many patients are found in stage III or IV and the 5-year survival rate for stage III–IV NPC after conventional radiotherapy alone was 66.4–71.3% [31]–[33]. Given the chemosensitivity of NPC, standard two-dimensional (2D) radiotherapy with concurrent CDDP followed by adjuvant CDDP and fluorouracil showed better outcome, but had severe toxicity and resistance [14], [34], [35]. Therefore, developing new therapeutic strategies to overcome drug-resistance is the key priority in NPC treatment. In our study, we found that the dual PI3K/mTOR inhibitor selectively inhibited NPC cells proliferation and suppressed the PI3K/mTOR activity both *in vivo* and *in vitro* (Fig. 1, Fig. 2, Fig. 6A–E). Furthermore, NVP-BEZ235 induced cell cycle arrest in G1 phase by down-regulating CDK4 and CyclinD1, up-regulating P21 and P27 (Fig. 3A and B). Also, we found that the PI3K/mTOR pathway was activated following CDDP treatment with increased phosphorylation of AKT, GSK3β, S6K and 4E-BP1 (Fig. 4A) and NVP-BEZ235 significantly alleviated the phosphorylation of these substrates (Fig. 4B). The AKT pathway is one of the most common molecular alterations in NPC which may be the reason of CDDP resistance [36], [37]. Therefore, the combination of CDDP and PI3K/mTOR inhibitor could synergistically increase the antitumor efficacy in NPC both *in vivo* and *in vitro* (Fig. 5, Fig. 6 A–D and F). This study is the first to provide evidence for the efficacy of the novel dual PI3K/mTOR inhibitor NVP-BEZ235 in preclinical models of NPC and it has a potential therapeutic value as treatment alone or in combination with CDDP targeting NPC.

NVP-BEZ235 is a new dual PI3K and mTOR inhibitor whose efficacy in advanced solid tumours is currently being evaluated in a phase I/II clinical trial. We therefore hypothesized that the inhibition of PI3K/mTOR by NVP-BEZ235 led to decreased cell proliferation in NPC for the previous study of LY294002 in NPC [38]. As expected, NVP-BEZ235 strongly decreased NPC proliferation *in vivo* and *in vitro*. This is in accordance with the results that LY294002 and wortmannin inhibited PI3K and had strong inhibition of cell proliferation in NPC cells [39]. However, Eichhorn reported that NVP-BEZ235 selectively targeted patients with tumors harboring activating mutations of PI3K, with higher doses for those individuals with PTEN loss [11]. In our study, treatment of NVP-BEZ235 in PIK3CA mutant cell lines inhibited proliferation in a low dose, whereas the same NVP-BEZ235 doses failed to completely abrogate AKT activity in cells without PIK3CA mutant, which needed higher doses, consistent with the above experience. Our data also showed that administering NVP-BEZ235 both *in vivo* and *in vitro* could abrogate the phosphorylation of AKT, GSK3β, S6K and 4E-BP1 via dually inhibiting PI3K and mTOR kinase in NPC, harbouring activating mutations of PI3K/AKT pathway. However, other studies reported that the absence of activating mutations of PI3K/AKT pathway did not preclude the response to NVP-BEZ235. They showed that NVP-BEZ235 was effective in cell lines and tumor models with many oncogenic pathway mutations, such as K-RAS and B-RAF [10], [40]. Our unpublished data also showed that RAS/ERK pathway was activated by NVP-BEZ235 treatment, so concomitant inhibition of the RAS/ERK pathway in this model might be necessary to achieve greater antitumor effects.

Cell cycle analysis revealed that decreased cell proliferation was due to the induction of G1 arrest and Western blotting showed that G1 arrest was due to the decreased expression of cyclin D1, CDK4 and increased expression of P21, P27. As a PI3K/mTOR inhibitor, NVP-BEZ235 strongly blocked the AKT kinase activity and the phosphorylation of GSK3β downstream of AKT, which made the above AKT function inefficient and thereby induced G1 arrest. This is explained by another important function of AKT in G1/S progression which is a positive regulation of mid- and late-G1-phase cyclin/CDK activity via phosphorylation and inactivation of CDK inhibitors P21 and P27 [41].

Currently, CDDP-based combination chemotherapy is regarded as the most effective standard regimen for NPC with metastasis, and the combination of CDDP and 5-FU is the first-line treatment, with a 66–78% response rate [42]. CDDP-based combination treatment includes CDDP and 5-FU, CDDP and CF, CDDP, BLM and 5-FU. In our study, although CDDP treatment was effective *in vivo* (Figure 6A–D), we found that CDDP induced the activation of AKT and mTORC1 pathways, which decreased the inhibitory rates of CDDP treatment. In despite of which mechanisms, NVP-BEZ235, as a dual PI3K and mTOR inhibitor, strongly inhibited the activation of AKT and mTORC1 pathways by CDDP, which synergized CDDP sensitivity of NPC treatment. This suggested that NVP-BEZ235 might be able to sensitize platinum agents and this effect was due to the inhibition of CDDP-induced AKT and mTORC1 activation, being consistent with other tumors identifying AKT activation by CDDP [43], [44]. Katz has reported that activation of AKT by CDDP is dependent of SRC, EGFR and PI3K, which can be placed in one signal transduction pathway, with EGFR and SRC upstream of PI3K in breast cancer [29]. It should be noted that we concentrated only on the inhibitory effect of NVP-BEZ235 on NPC and found a combination strategy of CDDP and NVP-BEZ235. In the future we would further identify the mechanism that CDDP activated PI3K/AKT and mTORC1 pathways. Many groups have reported that the activation of PI3K/AKT/mTOR pathway is involved in various events, such as the critical crosstalk between PI3K/AKT signaling pathway and ROS that is essential for IL-7-mediated T-ALL cell survival, feedback activation from inhibition of other signals including MAPK pathway, HER2 and Estrogen receptor [45]–[48].

*In vivo,* NVP-BEZ235 displayed a statistically significant antitumor activity and synergy with CDDP against CNE2 and HONE1 xenografts. The tumor growth inhibition of NVP-BEZ235 targeting NPC xenografts was dose-dependent, and the observed effect of the phosphorylation of AKT, GSK3β, S6K and 4E-BP1 correlated with the amount of compound presented in the tumor tissue. Due to the negative feedback of the phosphorylation of S6K1, inhibition of mTORC1activity alone results in the reactivation of the PI3K axis [49]. Exposure to a dual PI3K/mTOR inhibitor such as NVP-BEZ235 might therefore be sufficient to block the feedback loop. Furthermore, NVP-BEZ235 could regulate Bcl-2 family proteins which are pro- and antiapoptotic mitochondrial effectors in carcinogenesis and therapy resistance [50]. NVP-BEZ235 decreased the levels of antiapoptotic proteins Bcl-2, Mcl-1 and increased proapoptotic proteins Bax, Noxa, which suggested that NVP-BEZ235 could strongly modulate apoptosis in NPC xenografts. The combination of CDDP and NVP-BEZ235 synergistically enhanced the regulation of Bcl-2 family members by NVP-BEZ235, in consistent with cleavages of Caspase-3 and PARP following NVP-BEZ235 treatment and the combination with CDDP.

Taken together, the results from the study showed that NVP-BEZ235 has a dramatic potential of NPC therapy *in vivo* and *in vitro* through inducing cell cycle arrest and apoptosis. Furthermore, targeting PI3K/AKT and mTOR by NVP-BEZ235 sensitized antitumor effect of CDDP in NPC. Here we have reported that CDDP activates PI3K/AKT and mTORC1 pathways, which provides a combination strategy of chemotherapy and small molecular inhibitor targeting signal pathway. These findings suggest that the dual targeting PI3K and mTOR pathways is a better modality of targeted therapy for tumors that harbor activation of the PI3K/mTOR pathway, such as NPC. However, a major challenge in the clinical use of PI3K/AKT/mTOR pathway inhibitors is to identify patients who will likely respond to the treatment.

## Supporting Information

Figure S1 **Raw data of oncomutation panel mutation list report.** Oncogenic mutation profiling details seen in the Materials and methods.
(TIF)

Figure S2 **Average body weights of nude mice in (A) CNE2 xenografts and (B) HONE1 xenografts.** Animal body weight was measured and recorded every 2∼3 days during the treatment, then calculated the average value of per group.
(TIF)
