# Supplementary figures and images for: Dual Phosphoinositide 3-Kinase/Mammalian Target of Rapamycin Inhibitor NVP-BEZ235 Has a Therapeutic Potential and Sensitizes Cisplatin in Nasopharyngeal Carcinoma
Source: PLoS One. 2013 Mar 22;8(3):e59879. doi: 10.1371/journal.pone.0059879 (PMC3606339; doi:10.1371/journal.pone.0059879)

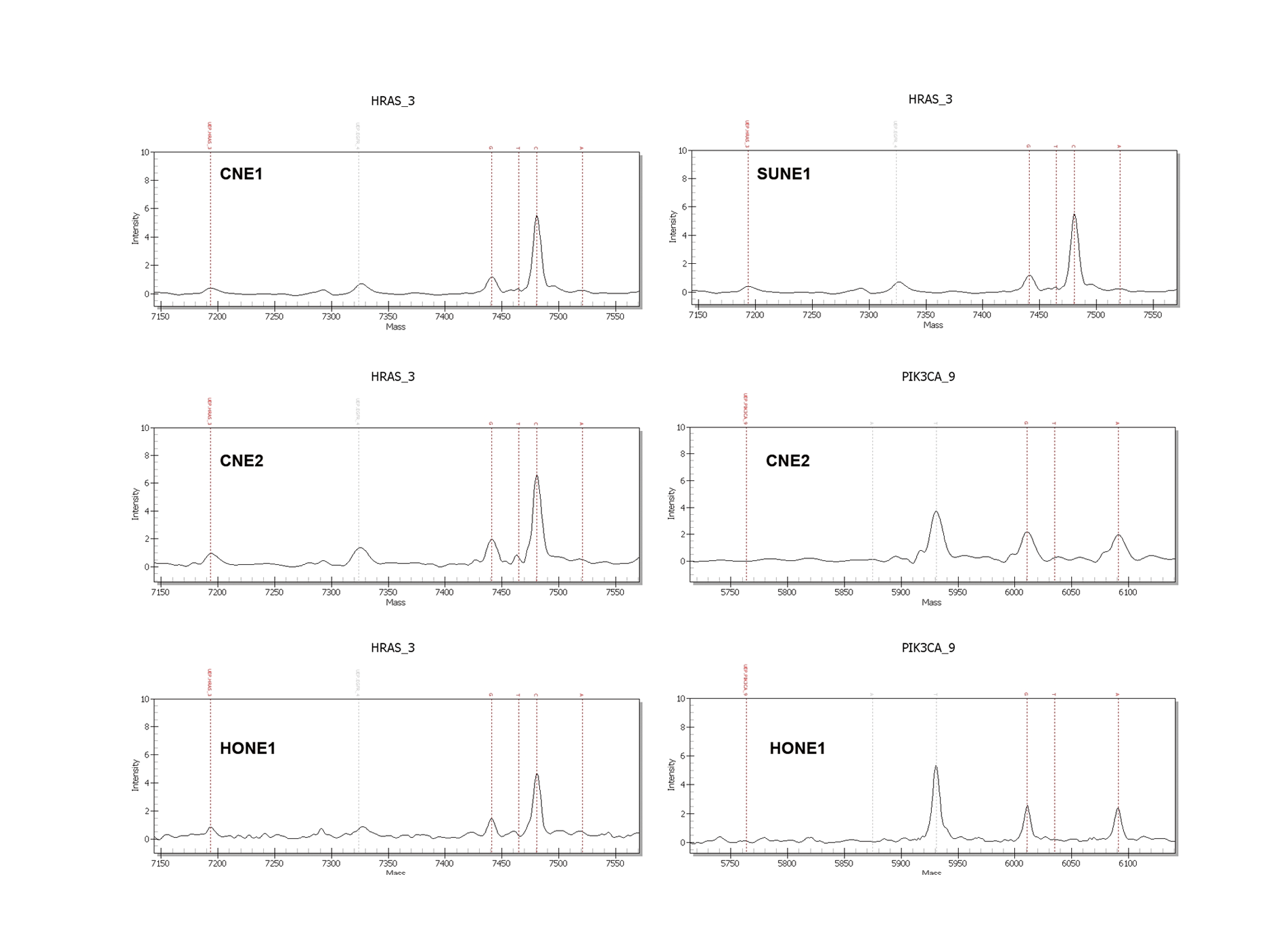

Supplement: Figure S1 — Raw data of oncomutation panel mutation list report. Oncogenic mutation profiling details seen in the Materials and methods. (TIF) [file pone.0059879.s001.tif]

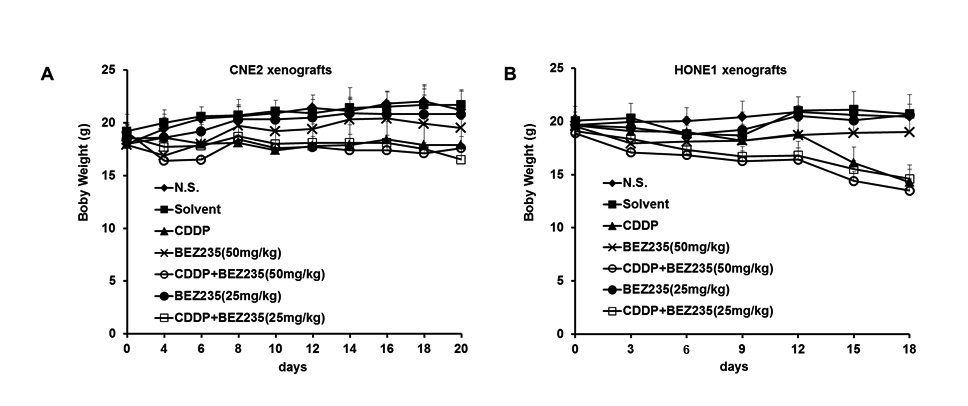

Supplement: Figure S2 — Average body weights of nude mice in (A) CNE2 xenografts and (B) HONE1 xenografts. Animal body weight was measured and recorded every 2∼3 days during the treatment, then calculated the average value of per group. (TIF) [file pone.0059879.s002.tif]
